# Supplementary material for: Exploring Predictors of Self-Perceived Cardiorespiratory Fitness ≥ 5 Years Beyond Breast Cancer Diagnosis: A Cross-Sectional Study
Source: Healthcare (Basel). 2025 Mar 24;13(7):718. doi: 10.3390/healthcare13070718 (PMC11988748; doi:10.3390/healthcare13070718)
Supplement: Supplementary file 1 [file healthcare-13-00718-s001.zip › healthcare-3513853-supplementary.pdf]

Supplementary Figure S1. Flow diagram for study participants

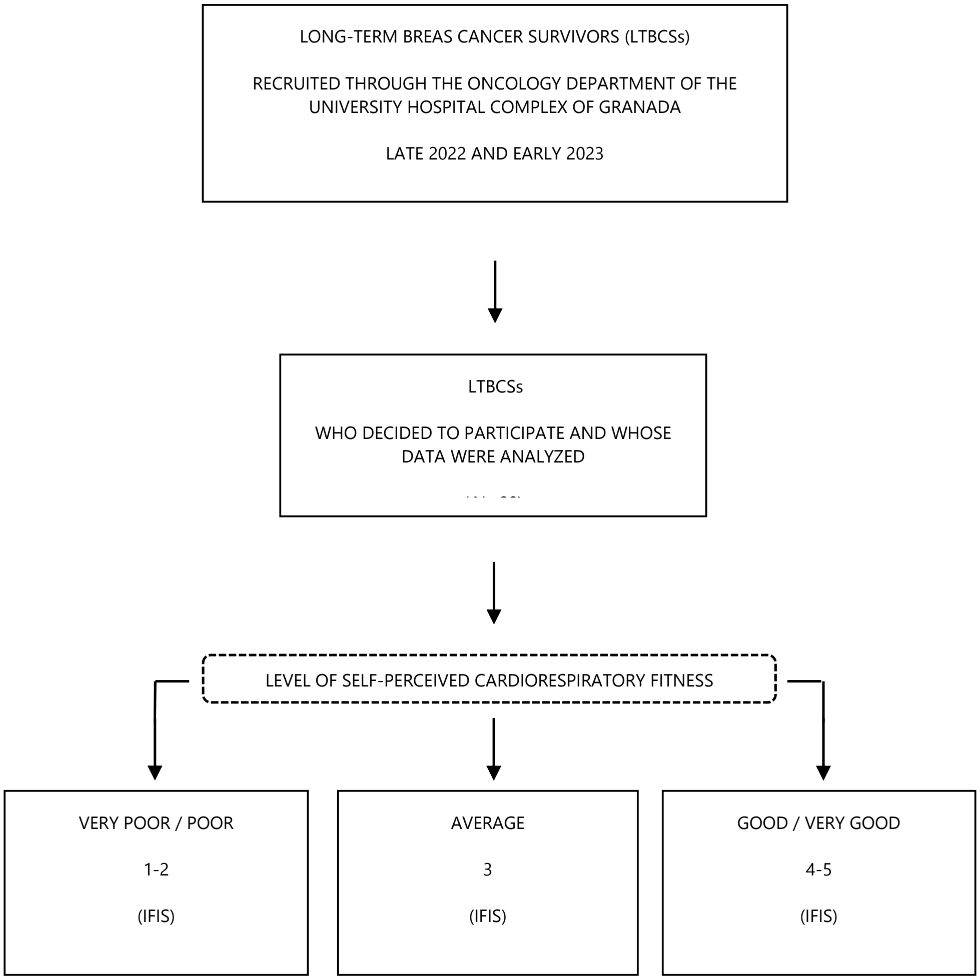

Abbreviations: LTBCS Long-term breast cancer survivors, IFIS International Fitness Scale, N/n simple size
